# The Prevention of House Dust Mite Allergies in Pediatric Asthma

**DOI:** 10.3390/children11040469

**Published:** 2024-04-15

**Authors:** Angela Klain, Antonio Andrea Senatore, Amelia Licari, Francesca Galletta, Irene Bettini, Leonardo Tomei, Sara Manti, Francesca Mori, Michele Miraglia del Giudice, Cristiana Indolfi

**Affiliations:** 1 Department of Woman, Child and General and Specialized Surgery, University of Campania ‘Luigi Vanvitelli’, 80138 Naples, Italy; angela.klain@studenti.unicampania.it (A.K.); cristiana.indolfi@policliniconapoli.it (C.I.); 2 Department of Clinical, Surgical, Diagnostic, and Pediatric Sciences, University of Pavia, 27100 Pavia, Italy; antonioandrea.senatore01@universitadipavia.it (A.A.S.); amelia.licari@unipv.it (A.L.); 3 Pediatric Clinic, Fondazione IRCCS Policlinico San Matteo, 27100 Pavia, Italy; 4 Pediatric Unit, Department of Human Pathology in Adult and Developmental Age ‘Gaetano Barresi’, University of Messina, 98122 Messina, Italy; francygall.92@gmail.com (F.G.); smanti@unime.it (S.M.); 5 Pediatric Unit, IRCCS Azienda Ospedaliera-Universitaria di Bologna, 40138 Bologna, Italy; irene.bettini@studio.unibo.it; 6 Allergy Unit, Meyer Children’s Hospital, IRCCS, 50139 Florence, Italy; leonardo.tomei@unifi.it (L.T.); francesca.mori@unifi.it (F.M.)

**Keywords:** house dust mite, allergy, asthma, children, prevention, environmental, measures

## Abstract

This review provides a concise overview of preventive measures against dust mite allergies in pediatric populations, emphasizing the need for a comprehensive and evolving approach. Dust mites, ubiquitous microscopic arachnids, pose a significant threat to children’s health, triggering allergies and asthma. Traditional preventive strategies such as regular cleaning, mattress covers, and humidity control are essential but warrant refinement. Empowering children through personalized hygiene education and exploring innovative bedding solutions showcase a forward-thinking paradigm. Collaboration with healthcare professionals and embracing technology-driven solutions ensures a holistic and adaptable approach to safeguarding pediatric health against dust mite-related ailments. This abstract underscores the importance of continually reassessing and innovating preventive measures to create resilient and health-conscious living environments for children.

## 1. Introduction

Dust mites, also known as house dust mites (HDMs), are a major source of environmental inhalant allergens, especially in temperate climates. In sensitized and genetically predisposed individuals, inhalation of these allergens can cause the onset or worsening of allergic diseases such as rhinitis and bronchial asthma [1]. The most relevant allergens in house dust are the fecal pellets of the *Dermatophagoides pteronyssinus* (Der p) species, primarily Der p 1, Der p 2, Der p 23, and *Dermatophagoides farinae* (Der f 1 and Der f 2). In temperate countries, over half of allergic individuals are sensitized to HDMs, and up to 85% of children with bronchial asthma are sensitized to these main Dermatophagoides species [2].

Studies have shown a link between HDM allergen levels in homes and asthma symptoms in children and adults sensitized to HDMs [3,4]. High exposure to these allergens at home can worsen asthma in HDM-sensitized patients, causing bronchospasms and increased bronchial hyperreactivity. Conversely, reducing exposure can improve symptoms [5]. While the evidence is not conclusive, some studies also suggest that reducing exposure in early life might decrease the risk of developing asthma in high-risk infants [6,7,8]. 

This suggests that mite avoidance appears to be a valuable approach for managing and potentially preventing asthma in children sensitized to HDMs. Several methods for reducing mite allergen exposure have been investigated [9]. By reviewing existing studies on this topic, this review aims to provide healthcare professionals with the most up-to-date information on preventing HDM allergies in children with asthma.

## 2. Understanding HDM Allergies and Asthma

Der p 1 and Der p 2 are the primary allergens of *Dermatophagoides pteronyssinus* because they are recognized by most subjects sensitized to the mite [10]. Der p 1 is a heat-labile glycoprotein present mainly in the mite’s fecal material, which is the main way in which mite allergens are dispersed in the home environment, and Der p 2, analogous to MD-2, LPS-binding component MD-2 of the Toll-like receptor 4 (TLR4) complex [10]. Recently, the Valenta group identified another major allergen: Der p 23, a peritrophic matrix protein that surrounds the mite’s feces [11]. This molecule is recognized by 74% of patients allergic to Der p 1 and Der p 2 and plays an important role both in the diagnosis of and in allergen immunotherapy (AIT) for patients allergic to mites [12,13]. HDM allergens are potent triggers of allergic sensitization and subsequent atopic respiratory diseases. These allergens are not limited to fecal particles but are also present in the mite’s gut, exoskeleton, and eggs [13]. Upon inhalation or contact with mucosal surfaces, including the epithelia of the upper and lower respiratory system, eyes, skin, and intestine, HDM allergens can induce sensitization. This can lead to the development of allergic rhinitis, conjunctivitis, sinusitis, and bronchial asthma [14]. Furthermore, mite proteases contribute directly to the pathogenesis of atopic dermatitis by causing epithelial damage through the disruption of tight junctions and the subsequent release of pro-inflammatory cytokines [15]. Interestingly, individuals sensitized to HDMs can also experience systemic allergic reactions following the ingestion of certain invertebrates, such as crustaceans. This phenomenon arises due to cross-reactivity at the molecular level between allergens present in crustaceans (e.g., tropomyosin Pen a 1, a major crustacean allergen) and HDM allergens (e.g., Der p 10). Sensitization to HDMs through inhalation can prime the immune system for such cross-reactions, leading to clinical manifestations upon exposure to crustacean allergens [16].

**Figure 1:**
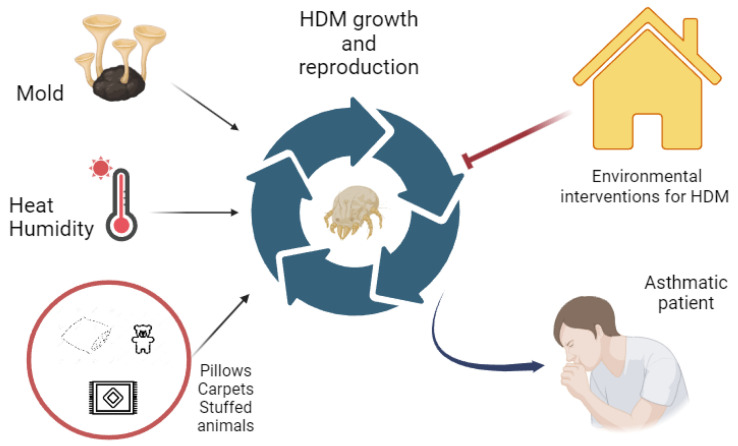
The relationship between environmental factors and the development of sensitization to house dust mites and bronchial asthma.

Although HDMs are mainly documented in the home environment, they can also be found, in varying quantities, in workplaces, in schools, and on means of transport [17]. A study conducted in the USA found that about half of homes had levels equal to or greater than the presumed allergic sensitization threshold (>2 μg/g) [18]. Although HDMs can adapt to survive in any closed environment and their allergens are found in reservoirs in various places in the home, such as furniture, toys, carpets, and upholstered materials, their main habitat is undoubtedly the bedroom (and especially in mattresses and pillows), as we spend most of our days there. In this specific ecosystem, mites find the environmental conditions suitable for their survival: adequate temperature and humidity, often poor lighting, and the presence of food sources represented mainly by human skin desquamation and food residues [1]. In recent decades, the progressive and sometimes radical changes in both home furnishings and our domestic habits have gradually led to a progressive increase in the proliferation of HDMs. Furthermore, the increasingly widespread use of heating means that in winter, the optimal temperature values for mite development are constantly reached. Similarly, the humidity of the internal air in micro-environments is often kept between 50 and 70%, also due to reduced ventilation, which decreases the dispersion of heat, causing a consequent increase in humidity. Another environmental factor that influences the quantitative presence of HDMs is altitude: it now seems well established that mites tend to disappear in high-mountain homes, if the altitude exceeds 1600 m [19] (Figure 1).

Different elements present in dust mite particles function as pathogen-associated molecular patterns (PAMPs), which attach to pattern recognition receptors (PRRs) found on epithelial cells and antigen-presenting cells, such as dendritic cells. Upon initial exposure, these PRRs identify PAMPs as belonging to foreign primitive organisms, triggering a Th2-directed immune response that produces IgE antibodies. Known PAMPs found in house dust mite feces and bodies encompass chitin, mite DNA, bacterial DNA, and endotoxin [20,21].

Chitin is a component of the exoskeletons of shrimp, crustaceans, insects, worms, and dust mites. This protein is highly allergenic, triggering the activation of the T2 immune system and the production of two asthma-related molecules: acidic mammalian chitinase (AMC) and the chitinase-related protein YKL-40 [1,22]. These proteins serve as markers of severe asthma in sensitized individuals. Additionally, endotoxins present in dust mite faeces act as allergens, inducing inflammation and hyper-reactivity in the bronchial pathways [1,23]. 

Several risk factors contribute to the development and exacerbation of HDM allergies in children with asthma [24]. These include the following: (i) genetic predisposition (children with a family history of allergies, asthma, or atopic dermatitis are at higher risk of developing HDM allergies and asthma); (ii) exposure to HDMs (prolonged exposure to HDMs, particularly in bedding, carpets, upholstered furniture, and stuffed toys, increases the risk of sensitization and exacerbation of asthma symptoms); (iii) early exposure during infancy or childhood, as this increases the likelihood of developing sensitization and asthma symptoms later in life; (iv) the indoor environment (i.e., poor indoor air quality, inadequate ventilation, and high humidity levels create favorable conditions for HDMs to thrive, increasing the risk of sensitization and asthma exacerbations); (v) geographical location (i.e., children living in regions with a higher prevalence of HDMs, such as humid and temperate climates, are at increased risk of developing allergies and asthma) [25]; (vi) poor housing conditions (i.e., crowded living spaces, lack of regular cleaning, and the presence of carpets or heavy drapes provide ideal habitats for HDMs, increasing exposure and the risk of sensitization); (vii) coexisting allergies (i.e., children with other allergic conditions, such as allergic rhinitis or eczema, are more likely to develop HDM allergies and experience asthma exacerbations triggered by HDMs); (viii) immune dysfunction, such as impaired regulatory T-cell function or altered cytokine profiles, as this may cause increased susceptibility to HDM allergies and asthma; and (iv) tobacco smoke exposure, either during pregnancy or after giving birth, which is associated with an increased risk of developing asthma and allergies, including HDM allergies [24]. 

## 3. Preventive Strategies for HDM Allergies

Effective preventive measures are critical for providing a safe and healthy environment for pediatric well-being. Prevention can be implemented across different levels. Primary prevention focuses on preventing the initial development of allergies in individuals who have not yet developed HDM allergic sensitization. This involves measures to minimize exposure to HDM allergens in susceptible individuals, particularly during critical periods of immune system development, such as early childhood. Secondary prevention aims to prevent the progression of allergic diseases in individuals who have already developed HDM sensitization but have not yet developed clinical symptoms such as asthma or allergic rhinitis. Tertiary prevention focuses on managing and minimizing the impact of established allergic diseases or asthma caused by HDM allergens. The avoidance of HDM allergens contributes to primary, secondary, and tertiary prevention. AIT represents a therapeutic option for patients with asthma or allergic rhinitis due to HDMs, and therefore it can be regarded as a tertiary prevention measure. AIT not only provides symptomatic relief but also aims to prevent the progression of allergic diseases and reduce the need for long-term pharmacological treatments. Finally, probiotics have been the subject of extensive research, particularly concerning tertiary prevention; nevertheless, their potential contribution to secondary or primary prevention is still being investigated and shows promise.

### 3.1. Environmental Modifications

Environmental modifications are crucial in managing allergens and promoting a healthier living space. The first environmental modification addresses the bedroom environment, where children spend most of their time. Encasing mattresses, pillows, and comforters can prevent common triggers for asthma symptoms, such as HDMs, from exacerbating respiratory issues in susceptible individuals. This involves the use of specially designed covers or encasements that act as a barrier, preventing HDMs from colonizing and thriving in bedding materials. The objective is to create a physical barrier that reduces an individual’s exposure to allergens, potentially alleviating allergic reactions and respiratory symptoms in individuals sensitive to these microscopic organisms [26]. The effectiveness of allergy-proof bedding lies in its construction. There are two types of textiles suitable for encasing materials: woven textiles and nonwoven textiles. Nonwoven fabrics are produced from fibers or filaments through mechanical, aerodynamic, or hydrodynamic processes. The resulting textiles exhibit effective filtration properties, though they may display variable layer thicknesses and surface irregularities. Woven textiles require a particularly tight weave, resulting in a consistently dense fabric. Additional processing, known as “finishing”, further reduces the pore size. The previous necessity of polyurethane coatings for encasings, prevalent in the past, is now obsolete due to the utilization of modern fibers and techniques [27]. The pore size, determined by the tightness of the weave, is crucial. Effective allergy-proof bedding, especially against dust mites, requires a pore size of 0.5 microns or less [28]. Laminates, often cost-effective, are waterproof, making them suitable for mattress protectors. Microfiber covers, on the other hand, tend to be cooler but may lack waterproofing [29]. Both materials require special care during washing, with laminates being sensitive to high temperatures and microfibers needing gentle washing without chlorine bleach. Vinyl is commonly used for box-spring covers.

Stuffed animals are often cherished companions for many children, but they can also harbor dust mites and contribute to allergen exposure. There are a few ways to eliminate these risks. One effective approach is to remove all stuffed animals from bedrooms or the entire house. Another option is to place the stuffed animals in a pillowcase or laundry bag and wash them in hot water, preferably at temperatures above 130 °F (54 °C), on a regular basis. Alternatively, they can be placed in a plastic bag and stored in the freezer for 24 h. After either method, one should make sure to thoroughly dry the stuffed animals, either by air-drying them or using a hot dryer [30].

Replacing carpets with hard floor surfaces, such as wood, linoleum, or tile, can also reduce allergen reservoirs and improve cleaning effectiveness, as carpets trap dust and allergens [31].

Washing bedding, including sheets and pillowcases, in hot water at least once a week is recommended to eliminate dust mites and other allergens that may accumulate over time. Washing in hot water at a temperature of at least 130 °F or 54 °C is effective in removing dust mites and their allergenic particles [32].

To manage asthma in children, especially those who are sensitized to HDMs, it is crucial to minimize clutter and dust traps in living spaces. Cluttered areas can harbor dust mites and allergens, worsening respiratory symptoms in children with asthma [32].

It is important to maintain humidity levels between 30 and 50% to manage house dust mite allergies because dust mites thrive in humid environments. Low humidity creates an inhospitable environment for dust mites, inhibiting their reproduction and growth. This also helps prevent mold development, which can trigger asthma symptoms [27]. The American Academy of Allergy, Asthma & Immunology (AAAAI) recommends using dehumidifiers in homes, especially in regions with high humidity, to create an environment less conducive to dust mite proliferation [33].

### 3.2. Air Filtration

High-Efficiency Particulate Air (HEPA) purifiers are effective in trapping airborne particles, including allergens such as HDM fecal particles and their fragments. The filter is the core component of an HEPA purifier, consisting of a dense mat of randomly arranged fibers made of fiberglass or other synthetic materials. To increase the surface area for capturing particles, the filter material is often pleated or folded, which enhances the overall efficiency of the purifier [34]. The filter is usually encased in a sturdy frame, which holds it in place and ensures a secure fit within the air purifier. To prevent air from bypassing the filter, HEPA purifiers often have seals and gaskets to ensure the air passes through the filter material, maximizing filtration efficiency. HEPA purifiers incorporate a fan or blower to draw air into the device, passing it through the HEPA filter, which helps circulate the purified air into the living space. Some HEPA purifiers include a pre-filter, which captures larger particles and helps prolong the life of the HEPA filter by preventing it from clogging quickly. By incorporating HEPA filters into a vacuuming routine, this structured cleaning process ensures the removal of a significant portion of airborne allergens and particulate matter from carpets and upholstery [35].

These environmental modifications collectively contribute to creating a healthier indoor environment while minimizing allergen exposure and improving the overall well-being of individuals susceptible to respiratory allergies.

### 3.3. Allergen Immunotherapy

Beyond environmental prophylaxis, AIT is currently considered the only therapeutic intervention capable of modifying the natural history of a disease for IgE-mediated diseases [36,37]. AIT is recommended for any patients who do not respond, or respond partially, to the usual allergen avoidance strategies or to pharmacological therapies, providing a clinical benefit that can persist for several years after the treatment is discontinued. AIT exerts its action essentially through an immunological mechanism aimed at promoting regulatory T-cells, “minimizing” the immune response induced by the allergens themselves [38]. 

Evidence supporting the use of AIT in children is well documented both for persistent bronchial asthma and, above all, for moderate to severe allergic rhinitis. For both pathologies, the significant efficacy of AIT has been documented in controlling and reducing the symptom complex, in ensuring the safety of drugs, and in reducing the progression of these pathologies to more severe forms [39,40]. AIT also exerts preventive action on the development of further new sensitizations.

In the context of HDM allergies, researchers have investigated both subcutaneous immunotherapy and sublingual immunotherapy (SLIT) to treat children and adolescents with allergic rhinitis and asthma [41]. However, subcutaneous immunotherapy carries a risk of serious and potentially fatal side effects for asthmatic patients, especially those with uncontrolled asthma. This concern makes SLIT an attractive alternative, particularly for pediatric patients, because its side effects are typically mild and localized, such as itching in the mouth and swelling of the lips or tongue [42,43]. 

A review of the literature by Richards et al. evaluated the administration of HDM SLIT in pediatric patients with allergic asthma [44]. Studies on HDM SLIT for children with allergic rhinitis and asthma caused by HDMs alone had mixed results, possibly due to differences in how the studies were conducted. While eight studies showed that SLIT reduced asthma symptoms compared to a placebo, only four showed a decrease in the need for asthma medication. Compared to just medication, both SLIT and SCIT seemed to improve asthma symptom scores for up to 3 years [44]. One recent meta-analysis confirmed that children with asthma and allergic rhinitis who received AIT showed a combined improvement in their asthma and rhinitis symptoms, medication use, and need for quick-relief medications [45]. These benefits even seemed to last after the AIT stopped. One study found that children with asthma who received HDM AIT experienced fewer asthma symptoms and showed less medication use (including inhalers), better lung function, and higher peak expiratory flow rates (a measure of lung function) five years after stopping treatment compared to a control group [46]. These consistent results should be interpreted considering variations due to different treatment types and how success was measured.

According to the most recent EAACI guidelines, only HDM SLIT tablets are recommended as an add-on treatment for adults with controlled or partly controlled asthma caused by HDM allergies (moderate-quality evidence, conditional recommendation). While HDM subcutaneous immunotherapy injections are also recommended for adults and children, and SLIT drops are an option for children with controlled HDM allergies, the evidence for their effectiveness in reducing symptoms and medication needs is less conclusive (low-quality evidence, conditional recommendation) [47].

### 3.4. Probiotics

Probiotics have emerged as a promising area of research surrounding pediatric asthma. Potential mechanisms through which probiotics exert their effects on asthma include modulating the gut microbiota and subsequent influence on the systemic immune response. Studies such as the one conducted by Arrieta et al. on mice, and those conducted by Aguanno et al. and Wu and colleagues on humans, have highlighted connections between the gut microbiota composition, immune development, and the prevalence of allergic diseases, providing insights into the role of probiotics in shaping a healthier immune profile in children with asthma [48,49,50]. 

Moreover, recent meta-analyses, such as the comprehensive review by Cuello-Garcia et al., have attempted to consolidate findings from multiple studies. This meta-analysis suggested that probiotic interventions might have a beneficial impact on asthma-related outcomes in children, supporting the idea that probiotics could be a valuable addition to the management strategies for pediatric asthma with house dust mite sensitizations [51].

Presently, the understanding of the “gut–lung axis” is progressively solidifying. The gut–lung axis is a concept that underscores the intricate interplay between the gastrointestinal tract and the respiratory system [52]. This bidirectional communication involves a network of immune responses, microbial interactions, and neural connections between the two systems. The gut is home to a diverse community of microorganisms collectively known as the microbiota. These microbes play a vital role in shaping immune responses. Disturbances in the gut microbial balance can impact lung health and contribute to respiratory diseases. This bidirectional influence suggests potential therapeutic interventions, such as probiotics and dietary modifications, to improve gut and lung health. In the context of pediatric dust mite allergies, specific probiotic strains are under scrutiny for their preventive and therapeutic capacities, like *Lactobacillus rhamnosus GG* (LGG), *Lactobacillus reuteri*, *Bifidobacterium breve*, *Bifidobacterium lactis*, *Lactobacillus casei*, and *Bifidobacterium infantis* [53]. Over the past decade, numerous studies have investigated the potential benefits of probiotics in managing asthma symptoms and addressing allergic sensitivities [54].

Recent research has explored the immunomodulatory effects of probiotics, aiming to understand their impact on the respiratory health of children with HDM sensitizations. In an investigation by Hisbergues et al., the co-administration of L. plantarum and Der p 1 led to a shift towards a Th1 immune response, characterized by an increased production of IgG and Th1 cytokines such as IFN-γ and IL-12. Additionally, there was a moderate increase in IL-10 production, a reduction in eosinophilia observed in the bronchoalveolar lavage following the allergen challenge, and a suppression of the allergen-specific IgE response [55]. In another study involving 29 patients with allergic asthma and HDM sensitization, a 4-week treatment with synbiotics significantly reduced the systemic production of Th2-cytokines, particularly IL-5, after an allergen challenge. Despite the absence of observable effects on bronchial inflammation markers, the treatment also led to an improvement in the peak expiratory flow [56]. In the study by Berings et al., covers infused with probiotics of the Bacillus subtilis, Bacillus amylo liquefaciens, and Bacillus pumilus strains were associated with a notable enhancement in various symptoms and allergy-related quality-of-life scores (considered as secondary outcomes) when comparing the covers infused with probiotics to the baseline. In contrast, such improvements were not observed with placebo covers [57]. Further research is warranted to elucidate the optimal strains, dosages, and duration of probiotic supplementation, paving the way for personalized and effective interventions for the management of HDM-sensitized children.

All of the preventive measures have been summarized in Table 1.

## 4. The Effects of Preventive Strategies for HDM Allergies on Pediatric Asthma 

The effects of preventive strategies are complex and mixed, depending on several factors. Implementing simple measures, such as decluttering and regular cleaning, effectively decreases dust mite exposure [9]. Several studies have validated that removing or regularly washing stuffed animals effectively reduces airborne HDM allergens, significantly decreasing their presence [58,59]. Also, consistent dusting and vacuuming significantly reduces airborne allergens [60], positively impacting respiratory outcomes in patients with asthma and dust mite sensitivities [61]. Moreover, the management of humidity levels in the home induces a notable reduction in the prevalence of dust mites and an improvement in asthma control [62,63]. In one 12-month study involving 40 mite-sensitive asthmatic subjects, homes fitted with mechanical ventilation achieved markedly lower humidity levels, resulting in decreased dust mite numbers and lower concentrations of Der p 1. Adding a high-efficiency vacuum cleaner further enhanced this effect, suggesting a potential improvement in lung function, though the subjects’ symptoms showed only a trend towards improvement [64]. In Anhui, China, another study collected 189 dust samples from households, schools, and hotels to assess dust mite prevalence. The breeding rates were 34.67% on floors and 20.18% in air-conditioning filters, with households having the highest breeding density (10/g) [65]. The study suggested that regular indoor hygiene practices and air-conditioning filter cleaning can reduce exposure to indoor allergens, which was corroborated by allergy sufferers’ awareness of this effect. In an RCT by Gehring et al. focused on high-risk children with allergic mothers, participants were randomly assigned to receive active mattress covers, placebo covers, or no intervention, and the outcomes were evaluated over 8 years. The objective was to evaluate the effectiveness of early intervention through the use of mite-impermeable mattress covers in reducing the participants’ exposure to house dust mite (HDM) allergens. By the age of 8, participants in the active mattress cover group showed lower levels of the HDM allergen Der f1 compared to those in the placebo group. Repeated measure analyses revealed a temporary reduction in the likelihood of asthma symptoms at age 2 among participants in the intervention group. Furthermore, a transient correlation was observed between a higher exposure to HDM allergens at 3 months of age and an increase in asthma symptoms [66]. In a year-long study of 60 children with asthma and HDM allergies, those using active mattress and pillow encasings exhibited a significant and persistent reduction in their HDM allergen concentrations. Only the active treatment group showed a substantial decrease in inhaled steroid doses, dropping from a mean of 408 to 227 microg/d. Significant differences in steroid dose reduction between the groups were observed at 9 and 12 months. After 1 year, over 73% of children in the active treatment group achieved a reduction of 50% or more in inhaled steroid doses, compared to 24% in the placebo group (*p* < 0.01), and experienced fewer asthma attacks and hospitalizations [67]. In research involving 52 adolescent and adult patients with allergic asthma, van den Bemt and colleagues found that “encasing” mattresses led to a noteworthy decrease in the amount of Der p1 on the mattresses. Additionally, there was a significant enhancement in the patients’ morning peak expiratory flow rates [68]. In a study assessing the efficacy of HEPA filters in reducing allergic respiratory symptoms, 32 patients with perennial rhinitis and/or asthma experienced, on average, a 70% decrease in particulate matter when using the HEPA filter. While no significant difference in the overall symptom scores was observed, during periods without respiratory infection, the last 2 weeks of each filter period showed distinct improvements in the total and individual symptoms, supporting the conclusion that HEPA filters can effectively reduce allergic respiratory symptoms [69].

Moreover, preventive strategies may reduce the risk of asthma exacerbations. In a study involving mite-sensitized asthmatic children, HDM-impermeable bedding significantly reduced the rates of emergency hospital admissions for asthma exacerbations by 45% compared to a placebo group. After a 12-month intervention, in the active group, 29.3% of children had attended the hospital, whereas 41.5% did in the placebo group (*p* = 0.047). The annual rate of emergency hospital attendance was 27% lower in the active group, although not statistically significant. However, no significant difference was observed in the use of oral prednisolone for exacerbations between the groups [58]. In a study involving 25 adults with moderate or severe atopic asthma, the use of microfine-fiber covers on pillows and mattresses, along with allergen-avoidance counseling, led to a reduction in Der p 1 allergen levels in the intervention patients’ bedrooms. This reduction was associated with significant improvements in their asthma symptom scores and an increase in the minimum % peak expiratory flow from 2009 to 2010. Control patients who did not receive special covers or counseling did not show these improvements [70]. In a randomized controlled trial involving 62 asthmatic children living in homes with indoor mold, those receiving both medical/behavioral interventions and constructional remediation aimed at the moisture sources in their homes showed a notable reduction in symptom days and decreased healthcare utilization compared to the control group. The remediation group exhibited improved asthma outcomes, highlighting the effectiveness of addressing the root cause of moisture issues in homes with documented mold problems [71].

Reduced symptoms and the potential for fewer asthma attacks can translate to better sleep, more participation in activities, and an overall improved quality of life for children with asthma. Recent work by James et al. investigated the long-term effects of HEPA-filtered vacuuming on the overall quality of life of children with asthma and HDM sensitivities. The results revealed a notable decrease in respiratory symptoms and an improvement in sleep quality, highlighting the broader impact of this intervention beyond immediate respiratory outcomes [72].

Despite the numerous recommendations regarding environmental prevention measures in HDM-sensitized children, the evidence is sometimes inconclusive due to inconsistency across studies, difficulties in isolating the HDM-specific effects from other asthma management strategies and external factors, and limited long-term data. The inconsistency of the studies might be due to factors like variations in the study design, intervention intensity, and participant characteristics. The study by Koopman et al. investigated the effects of house dust mite (HDM) allergen avoidance on respiratory symptoms, atopic dermatitis, and sensitization in 1282 allergic pregnant women. Those who received HDM-allergen-impermeable mattress covers exhibited a reduced prevalence of nighttime coughing without a cold in the second year of their child’s life. However, no significant effects were observed on other respiratory symptoms, atopic dermatitis, or immunoglobulin E levels, suggesting a limited short-term impact with potential long-term effects yet to be determined [73]. In a study involving high-risk children, prenatal recruitment, and random allocation to mite-impermeable mattress covers or placebos/no intervention, the intervention successfully lowered Der f1 HDM allergen levels at age 8, with a temporary reduction in asthma symptoms at age 2. Nevertheless, this early intervention did not reduce the risk of hay fever, eczema, or allergic sensitization, indicating limited long-term protective effects beyond the temporary reduction in asthma symptoms [66]. In the study conducted by Koh and colleagues, a correlation was observed between floor vacuuming and heightened sensitization to dust mite allergens, as well as elevated levels of an atopy biomarker [74]. Two large birth cohort studies have investigated the effects of environmental allergens, including dust mites, on the development of allergies. In the Prevention and Incidence of Asthma and Mite Allergy (PIAMA) birth cohort study, the utilization of mite-impermeable mattress covers among infants at high risk was linked to a reduced likelihood of experiencing asthma symptoms at 2 years old. However, this association was not observed at other ages up to 8 years, and it did not lower the risk of hay fever, eczema, or allergic sensitization up to age 8 [73]. In the Manchester Asthma and Allergy Study (MAAS), there was no observed reduction in the prevalence of wheezing, nocturnal coughing unrelated to colds, eczema, or sensitization to HDM allergens in the active group compared to the control group. However, the active group exhibited a lower prevalence of severe respiratory manifestations, such as recurrent wheezing with shortness of breath and the need for medication prescriptions for wheezing, compared to the control group [75]. 

In the study by Woodcock et al. regarding the influence of early exposure to allergens, including HDMs, on the development of allergies and asthma in children, the findings indicated that overly reducing allergen exposure during the early years of life elevates the risks of allergic sensitization and asthma [76]. A potential explanation for this may lie in the “hygiene hypothesis”. The modern concept of the hygiene hypothesis proposes that reduced exposure to microbes and allergens early in life due to improved hygiene practices may lead to an increased risk of developing allergies and asthma. This theory challenges the traditional belief that cleanliness is always beneficial for health. According to this hypothesis, a lack of exposure to diverse microbial and environmental stimuli during critical periods of immune system development can disrupt the natural balance of immune responses, leading to an increased susceptibility to allergic diseases [77]. The hygiene hypothesis is an important concept to consider in the context of allergen sensitization, particularly for the sensitization to HDMs. Epidemiological studies have shown that early exposure to a wide range of microbes and allergens, including HDMs, can help modulate the immune system and reduce susceptibility to allergic diseases [78,79]. Therefore, in the context of preventing allergen sensitization, it is important to consider the hygiene hypothesis and promote an environment that encourages a moderate and diversified exposure to allergens during early childhood. This may include strategies such as exposure to pets, exposure to a variety of foods, and less stringent cleaning of the home environment [80,81]. Furthermore, the development of HDM sensitization and asthma is influenced by a complex interplay of genetic, environmental, and lifestyle factors, which may result in a reduction in the effectiveness of prevention measures. Genetic predisposition plays a significant role in determining an individual’s susceptibility to HDM sensitization and asthma. Numerous studies have identified genetic variants associated with an increased risk of developing allergic diseases, including genes involved in immune regulation, such as those encoding cytokines, immunoglobulins, and receptors involved in allergic inflammation [82]. Additionally, genetic factors, including members of the endoplasmic reticulum, may influence the responsiveness to HDM allergens and the severity of asthma symptoms [83,84,85]. Lifestyle choices and behaviors also play a role in modulating the risk of HDM sensitization and asthma. Diet, physical activity, smoking, and exposure to pollutants can influence immune function and respiratory health. For example, diets rich in fruits, vegetables, and omega-3 fatty acids have been associated with a reduced risk of allergic diseases, while high-fat and processed diets may exacerbate inflammation [86]. Regular physical activity has been shown to improve lung function and reduce the severity of asthma symptoms [87,88]. Conversely, exposure to tobacco smoke, air pollution, and occupational allergens can increase the risk of asthma development and worsen symptoms in individuals sensitized to HDM allergens [89].

In most studies on the clinical effectiveness of anti-dust-mite prevention measures, most benefits come from combining several interventions rather than utilizing a single strategy. One recent meta-analysis emphasized the importance of multifaceted approaches in managing asthma triggered by HDM allergens. Encasing bedding materials was identified as a key component, complementing other strategies such as regularly washing bedding in hot water and minimizing dust reservoirs in the bedroom [9,90,91]. The study by Arshad et al. emphasized the importance of properly implementing HEPA air purifiers in homes with asthmatic children. This includes choosing the appropriate sizes of purifiers for the room, the regular maintenance of their filters, and strategic placement to maximize their effectiveness [59]. While primary prevention studies lack consistent evidence on allergen avoidance, recent trials suggest the benefits of a pragmatic clinical approach: personalized interventions based on sensitization and exposure, multifaceted allergen control, and early intervention for childhood asthma management [92]. The effectiveness of preventive measures might be more significant for individuals with severe asthma and confirmed HDM allergies. Given the heightened sensitivity of severely asthmatic patients to specific triggers, implementing targeted interventions becomes essential for managing symptoms and minimizing exacerbations. This is especially relevant for those with confirmed HDM allergies, as ongoing exposure poses a continuous risk, necessitating stringent preventive strategies. Adhering to these measures becomes paramount for this subgroup, considering their susceptibility to severe consequences of allergen exposure. Moreover, implementing certain strategies to address asthma triggers can be financially burdensome and may necessitate continuous effort, raising practical concerns for many families. The cost and sustained commitment involved in, for example, maintaining allergen-free environments or installing specialized ventilation systems may limit the accessibility of such measures. Balancing the effectiveness of these strategies with their feasibility for diverse households is crucial to ensure equitable access to asthma management resources. 

Addressing these practical considerations is essential for developing comprehensive and inclusive asthma prevention and control approaches.

While the evidence is still evolving, preventive strategies for HDM allergies can be a valuable tool for managing childhood asthma, especially when combined with other asthma management approaches. However, more research is needed to fully understand their long-term effectiveness and cost-effectiveness in diverse populations. 

## 5. Conclusions

HDM allergies are a major culprit for childhood asthma. By preventing exposure to these allergens, we can significantly improve the quality of life of children with asthma and potentially reduce the severity of their asthma.

In a combined, long-term approach, multiple strategies, such as environmental modifications and HDM AIT, should be integrated to maximize the suppression of HDM allergens and desensitize children, ultimately improving their asthma control and quality of life. While these strategies are effective, there is always room for improvement. Continued research into early intervention and developing even more effective prevention methods are crucial. Additionally, raising awareness among parents, healthcare professionals, and the general public about the link between HDM allergies and childhood asthma can empower individuals to take action.

By combining preventative measures, promoting long-term commitment, and fostering ongoing research and education, we can significantly reduce the burden of HDM-allergy-induced asthma in children.

## Figures and Tables

**Table 1:** Preventative measures for house dust mites and their objectives.

Preventive Measure	Objectives
Environmental modifications	Promote a healthier living space and manage allergens by addressing the bedroom environment, encasing mattresses, pillows, and comforters to prevent triggers for asthma symptoms, eliminating dust mites from stuffed animals, replacing carpets with hard floor surfaces, washing bedding regularly in hot water, minimizing clutter, maintaining humidity levels between 30 and 50%, and using dehumidifiers.
Air filtration	Utilize High-Efficiency Particulate Air (HEPA) purifiers to trap airborne allergens, incorporating HEPA filters into vacuuming routines, and improving overall indoor air quality.
Allergen immunotherapy	Alter the course of IgE-mediated diseases, such as asthma and allergic rhinitis, by promoting regulatory T-cells and preventing new sensitizations through allergen immunotherapy (AIT) interventions, including sublingual immunotherapy (SLIT) and subcutaneous immunotherapy (SCIT).
Probiotics	Explore the potential of probiotics in modulating the gut microbiota, shaping immune responses, and improving asthma-related outcomes in children sensitized to house dust mites.

## Data Availability

Not applicable.

## References

[1] Miller J.D. (2019). The Role of Dust Mites in Allergy. Clin. Rev. Allergy Immunol..

[2] Peat J.K., Tovey E., Toelle B.G., Haby M.M., Gray E.J., Mahmic A., Woolcock A.J. (1996). House dust mite allergens. A major risk factor for childhood asthma in Australia. Am. J. Respir. Crit. Care Med..

[3] Chan-Yeung M., Manfreda J., Dimich-Ward H., Lam J., Ferguson A., Warren P., Simons E., Broder I., Chapman M., Platts-Mills T. (1995). Mite and cat allergen levels in homes and severity of asthma. Am. J. Respir. Crit. Care Med..

[4] Custovic A., Taggart S.C., Francis H.C., Chapman M.D., Woodcock A. (1996). Exposure to house dust mite allergens and the clinical activity of asthma. J. Allergy Clin. Immunol..

[5] Platts-Mills T.A., Tovey E.R., Mitchell E.B., Moszoro H., Nock P., Wilkins S.R. (1982). Reduction of bronchial hyperreactivity during prolonged allergen avoidance. Lancet.

[6] Gabet S., Rancière F., Just J., de Blic J., Lezmi G., Amat F., Seta N., Momas I. (2019). Asthma and allergic rhinitis risk depends on house dust mite specific IgE levels in PARIS birth cohort children. World Allergy Organ. J..

[7] Lebold K.M., Drake M.G., Pincus A.B., Pierce A.B., Fryer A.D., Jacoby D.B. (2022). Unique Allergic Asthma Phenotypes in Offspring of House Dust Mite-exposed Mice. Am. J. Respir. Cell Mol. Biol..

[8] Su K.W., Chiu C.Y., Tsai M.H., Liao S.L., Chen L.C., Hua M.C., Yao T.C., Huang J.L., Yeh K.W. (2019). Asymptomatic toddlers with house dust mite sensitization at risk of asthma and abnormal lung functions at age 7 years. World Allergy Organ. J..

[9] Zuiani C., Custovic A. (2020). Update on House Dust Mite Allergen Avoidance Measures for Asthma. Curr. Allergy Asthma Rep..

[10] Dramburg S., Hilger C., Santos A.F., de Las Vecillas L., Aalberse R.C., Acevedo N., Aglas L., Altmann F., Arruda K.L., Asero R. (2023). EAACI Molecular Allergology User’s Guide 2.0. Pediatr. Allergy Immunol..

[11] Weghofer M., Grote M., Resch Y., Casset A., Kneidinger M., Kopec J., Thomas W.R., Fernández-Caldas E., Kabesch M., Ferrara R. (2013). Identification of Der p 23, a peritrophin-like protein, as a new major Dermatophagoides pteronyssinus allergen associated with the peritrophic matrix of mite fecal pellets. J. Immunol..

[12] Banerjee S., Weber M., Blatt K., Swoboda I., Focke-Tejkl M., Valent P., Valenta R., Vrtala S. (2014). Conversion of Der p 23, a new major house dust mite allergen, into a hypoallergenic vaccine. J. Immunol..

[13] Bessot J.C., Pauli G. (2011). Mite allergens: An overview. Eur. Ann. Allergy Clin. Immunol..

[14] Louisias M., Ramadan A., Naja A.S., Phipatanakul W. (2019). The Effects of the Environment on Asthma Disease Activity. Immunol. Allergy Clin. N. Am..

[15] Langan S.M., Williams H.C. (2006). What causes worsening of eczema? A systematic review. Br. J. Dermatol..

[16] Wong L., Huang C.H., Lee B.W. (2016). Shellfish and House Dust Mite Allergies: Is the Link Tropomyosin?. Allergy Asthma Immunol. Res..

[17] Adgate J.L., Ramachandran G., Cho S.J., Ryan A.D., Grengs J. (2008). Allergen levels in inner city homes: Baseline concentrations and evaluation of intervention effectiveness. J. Expo. Sci. Environ. Epidemiol..

[18] Permaul P., Hoffman E., Fu C., Sheehan W., Baxi S., Gaffin J., Lane J., Bailey A., King E., Chapman M. (2012). Allergens in urban schools and homes of children with asthma. Pediatr. Allergy Immunol..

[19] Acevedo N., Zakzuk J., Caraballo L. (2019). House Dust Mite Allergy Under Changing Environments. Allergy Asthma Immunol. Res..

[20] Jacquet A., Robinson C. (2020). Proteolytic, lipidergic and polysaccharide molecular recognition shape innate responses to house dust mite allergens. Allergy.

[21] Jacquet A. (2011). The role of the house dust mite-induced innate immunity in development of allergic response. Int. Arch. Allergy Immunol..

[22] Trompette A., Divanovic S., Visintin A., Blanchard C., Hegde R.S., Madan R., Thorne P.S., Wills-Karp M., Gioannini T.L., Weiss J.P. (2009). Allergenicity resulting from functional mimicry of a Toll-like receptor complex protein. Nature.

[23] Dzoro S., Mittermann I., Resch-Marat Y., Vrtala S., Nehr M., Hirschl A.M., Wikberg G., Lundeberg L., Johansson C., Scheynius A. (2018). House dust mites as potential carriers for IgE sensitization to bacterial antigens. Allergy.

[24] Aggarwal P., Senthilkumaran S. (2023). Dust Mite Allergy. StatPearls [Internet].

[25] Posa D., Perna S., Resch Y., Lupinek C., Panetta V., Hofmaier S., Rohrbach A., Hatzler L., Grabenhenrich L., Tsilochristou O. (2017). Evolution and predictive value of IgE responses toward a comprehensive panel of house dust mite allergens during the first 2 decades of life. J. Allergy Clin. Immunol..

[26] Della Giustina A., Pattini S., Travaglini A., Brighetti M.A., Malizia V., Sfika V., Di Menno Di Bucchianico A., Tripodi S. (2023). Gli acari (della polvere) tra ieri, oggi e domani. Riv. Immunol. Allergol. Pediatr..

[27] Bemt L., Vries M.P., Knapen L., Jansen M., Goossens M., Muris J.W., Schayck C.P. (2006). Influence of mattress characteristics on house dust mite allergen concentration. Clin. Exp. Allergy.

[28] Klimek L., Brehler R., Bergmann K.C., Casper I., Klimek F., Hagemann J., Polk M.L., Cuevas M. (2023). Avoidance measures for mite allergy—An update. Allergo J. Int..

[29] Mahakittikun V., Boitano J.J., Tovey E., Bunnag C., Ninsanit P., Matsumoto T., Andre C. (2006). Mite penetration of different types of material claimed as mite proof by the Siriraj chamber method. J. Allergy Clin. Immunol..

[30] Carrard A., Pichler C. (2012). Hausstaubmilbenallergie [House dust mite allergy]. Ther. Umsch..

[31] Cinteza M., Daian C. (2014). House dust mite—The paradox. Maedica.

[32] McDonald L.G., Tovey E. (1992). The role of water temperature and laundry procedures in reducing house dust mite populations and allergen content of bedding. J. Allergy Clin. Immunol..

[33] American Academy of Allergy, Asthma & Immunology Managing Indoor Allergen Culprits. https://www.aaaai.org/tools-for-the-public/conditions-library/allergies/managing-indoor-allergen.

[34] Yu C.H., Yiin L.M., Tina Fan Z.H., Rhoads G.G. (2009). Evaluation of HEPA vacuum cleaning and dry steam cleaning in reducing levels of polycyclic aromatic hydrocarbons and house dust mite allergens in carpets. J. Environ. Monit..

[35] Munir A.K., Einarsson R., Dreborg S.K. (1993). Vacuum cleaning decreases the levels of mite allergens in house dust. Pediatr. Allergy Immunol..

[36] Pajno G.B., Bernardini R., Peroni D., Arasi S., Martelli A., Landi M., Passalacqua G., Muraro A., La Grutta S., Fiocchi A. (2017). Allergen-specific Immunotherapy panel of the Italian Society of Pediatric Allergy and Immunology (SIAIP). Clinical practice recommendations for allergen-specific immunotherapy in children: The Italian consensus report. Ital. J. Pediatr..

[37] Schmidlin K.A., Bernstein D.I. (2023). Safety of allergen immunotherapy in children. Curr. Opin. Allergy Clin. Immunol..

[38] Kucuksezer U.C., Ozdemir C., Cevhertas L., Ogulur I., Akdis M., Akdis C.A. (2020). Mechanisms of allergen-specific immunotherapy and allergen tolerance. Allergol. Int..

[39] Roberts G., Pfaar O., Akdis C.A., Ansotegui I.J., Durham S.R., Gerth van Wijk R., Halken S., Larenas-Linnemann D., Pawankar R., Pitsios C. (2018). EAACI Guidelines on Allergen Immunotherapy: Allergic rhinoconjunctivitis. Allergy.

[40] Dhami S., Kakourou A., Asamoah F., Agache I., Lau S., Jutel M., Muraro A., Roberts G., Akdis C.A., Bonini M. (2017). Allergen immunotherapy for allergic asthma: A systematic review and meta-analysis. Allergy.

[41] Tosca M.A., Olcese R., Licari A., Ciprandi G. (2020). Allergen immunotherapy and asthma. Pediatr. Allergy Immunol..

[42] Zheng C., Xu H., Huang S., Chen Z. (2023). Efficacy and safety of subcutaneous immunotherapy in asthmatic children allergic to house dust mite: A meta-analysis and systematic review. Front. Pediatr..

[43] Passalacqua G., Nowak-Wegrzyn A., Canonica G.W. (2017). Local side effects of sublingual and oral immunotherapy. J. Allergy Clin. Immunol. Pract..

[44] Richards J.R., Stumpf J.L. (2018). House Dust Mite Sublingual Immunotherapy for Pediatric Patients with Allergic Asthma. Ann. Pharmacother..

[45] Lin C.F., Lin Y.T., Liao C.K., Yeh T.H. (2023). Recent Updates of Immunotherapy for Allergic Rhinitis in Children. Curr. Otorhinolaryngol. Rep..

[46] Blanco C., Bazire R., Argiz L., Hernández-Peña J. (2018). Sublingual allergen immunotherapy for respiratory allergy: A systematic review. Drugs Context.

[47] Agache I., Lau S., Akdis C.A., Smolinska S., Bonini M., Cavkaytar O., Flood B., Gajdanowicz P., Izuhara K., Kalayci O. (2019). EAACI Guidelines on Allergen Immunotherapy: House dust mite-driven allergic asthma. Allergy.

[48] Arrieta M.C., Walter J., Finlay B.B. (2016). Human Microbiota-Associated Mice: A Model with Challenges. Cell Host Microbe.

[49] Aguanno D., Metwaly A., Coleman O.I., Haller D. (2022). Modeling microbiota-associated human diseases: From minimal models to complex systems. Microbiome Res. Rep..

[50] Wu D., Zhang Y., Dong S., Zhong C. (2021). Mutual interaction of microbiota and host immunity during health and diseases. Biophys. Rep..

[51] Cuello-Garcia C.A., Brożek J.L., Fiocchi A., Pawankar R., Yepes-Nuñez J.J., Terracciano L., Gandhi S., Agarwal A., Zhang Y., Schünemann H.J. (2015). Probiotics for the prevention of allergy: A systematic review and meta-analysis of randomized controlled trials. J. Allergy Clin. Immunol..

[52] Klain A., Dinardo G., Indolfi C., Contieri M., Salvatori A., Vitale S., Decimo F., Ciprandi G., Miraglia del Giudice M. (2023). Efficacy and Safety of Oral Probiotics in Children with Allergic Rhinitis: A Review. Allergies.

[53] Chiu C.Y., Chan Y.L., Tsai M.H., Wang C.J., Chiang M.H., Chiu C.C., Su S.C. (2020). Cross-talk between airway and gut microbiome links to IgE responses to house dust mites in childhood airway allergies. Sci. Rep..

[54] Fassio F., Guagnini F. (2018). House dust mite-related respiratory allergies and probiotics: A narrative review. Clin. Mol. Allergy.

[55] Hisbergues M., Magi M., Rigaux P., Steuve J., Garcia L., Goudercourt D., Pot B., Pestel J., Jacquet A. (2007). In vivo and in vitro immunomodulation of der p 1 allergen- specific response by Lactobacillus plantarum bacteria. Clin. Exp. Allergy.

[56] Van De Pol M.A., Lutter R., Smids B.S., Weersink E.J.M., Van Der Zee J.S. (2011). Synbiotics reduce allergen-induced T-helper 2 response and improve peak expiratory flow in allergic asthmatics. Allergy.

[57] Berings M., Jult A., Vermeulen H., De Ruyck N., Derycke L., Ucar H., Ghekiere P., Temmerman R., Ellis J., Bachert C. (2017). Probiotics-impregnated bedding covers for house dust miteallergic rhinitis: A pilot randomized clinical trial. Clin. Exp. Allergy.

[58] Murray C.S., Foden P., Sumner H., Shepley E., Custovic A., Simpson A. (2017). Preventing Severe Asthma Exacerbations in Children: A Randomized Trial of Mite-Impermeable Bedcovers. Am. J. Respir. Crit. Care Med..

[59] Arshad S.H., Bojarskas J., Tsitoura S., Matthews S., Mealy B., Dean T., Karmaus W., Frischer T., Kuehr J., Forster J. (2002). Prevention of sensitization to house dust mite by allergen avoidance in school-age children: A randomized controlled study. Clin. Exp. Allergy.

[60] Wu F.F., Wu M.W., Pierse N., Crane J., Siebers R. (2012). Daily vacuuming of mattresses significantly reduces house dust mite allergens, bacterial endotoxin, and fungal β-glucan. J. Asthma.

[61] van Boven F.E., de Jong N.W., Braunstahl G.J., Arends L.R., Gerth van Wijk R. (2020). Effectiveness of the Air Purification Strategies for the Treatment of Allergic Asthma: A Meta-Analysis. Int. Arch. Allergy Immunol..

[62] Hui-Beckman J.W., Goleva E., Leung D.Y.M., Kim B.E. (2023). The impact of temperature on the skin barrier and atopic dermatitis. Ann. Allergy Asthma Immunol..

[63] Rodriguez G.E., Branch L.B., Cotton E.K. (1975). The use of humidity in asthmatic children. J. Allergy Clin. Immunol..

[64] Warner J.A., Frederick J.M., Bryant T.N., Weich C., Raw G.J., Hunter C., Stephen F.R., McIntyre D.A., Warner J.O. (2000). Mechanical ventilation and high-efficiency vacuum cleaning: A combined strategy of mite and mite allergen reduction in the control of mite-sensitive asthma. J. Allergy Clin. Immunol..

[65] Xue Q., Zou M., Guo J., Teng Q., Zhang Q., Sheng L., Xu S., Fang C., Yao N., Li Y. (2023). Detection and assessment of dust mite allergens in an indoor environment in Anhui, China. Environ. Sci. Pollut. Res. Int..

[66] Gehring U., de Jongste J.C., Kerkhof M., Oldewening M., Postma D., van Strien R.T., Wijga A.H., Willers S.M., Wolse A., Gerritsen J. (2012). The 8-year follow-up of the PIAMA intervention study assessing the effect of mite-impermeable mattress covers. Allergy.

[67] Halken S., Høst A., Niklassen U., Hansen L.G., Nielsen F., Pedersen S., Osterballe O., Veggerby C., Poulsen L.K. (2003). Effect of mattress and pillow encasings on children with asthma and house dust mite allergy. J. Allergy Clin. Immunol..

[68] van den Bemt L., van Knapen L., de Vries M.P., Jansen M., Cloosterman S., van Schayck C.P. (2004). Clinical effectiveness of a mite allergen-impermeable bed-covering system in asthmatic mite-sensitive patients. J. Allergy Clin. Immunol..

[69] Reisman R.E., Mauriello P.M., Davis G.B., Georgitis J.W., DeMasi J.M. (1990). A double-blind study of the effectiveness of a high-efficiency particulate air (HEPA) filter in the treatment of patients with perennial allergic rhinitis and asthma. J. Allergy Clin. Immunol..

[70] Tsurikisawa N., Saito A., Oshikata C., Nakazawa T., Yasueda H., Akiyama K. (2013). Encasing bedding in covers made of microfine fibers reduces exposure to house mite allergens and improves disease management in adult atopic asthmatics. Allergy Asthma Clin. Immunol..

[71] Kercsmar C.M., Dearborn D.G., Schluchter M., Xue L., Kirchner H.L., Sobolewski J., Greenberg S.J., Vesper S.J., Allan T. (2006). Reduction in asthma morbidity in children due to home remediation aimed at moisture sources. Environ. Health Perspect..

[72] James C., Bernstein D.I., Cox J., Ryan P., Wolfe C., Jandarov R., Newman N., Indugula R., Reponen T. (2020). HEPA filtration improves asthma control in children exposed to traffic-related airborne particles. Indoor Air.

[73] Koopman L.P., van Strien R.T., Kerkhof M., Wijga A., Smit H.A., de Jongste J.C., Gerritsen J., Aalberse R.C., Brunekreef B., Neijens H.J. (2002). Placebo-controlled trial of house dust mite-impermeable mattress covers: Effect on symptoms in early childhood. Am. J. Respir. Crit. Care Med..

[74] Koh G.C., Shek L.P., Kee J., Tai B.C., Wee A., Ng V., Koh D. (2009). An association between floor vacuuming, dust-mite, and serum eosinophil cationic protein in young asthmatics. Indoor Air.

[75] Custovic A., Simpson B.M., Simpson A., Hallam C., Craven M., Brutsche M., Woodcock A. (2000). Manchester Asthma and Allergy Study: Low-allergen environment can be achieved and maintained during pregnancy and in early life. J. Allergy Clin. Immunol..

[76] Woodcock A., Lowe L.A., Murray C.S., Simpson B.M., Pipis S.D., Kissen P., Simpson A., Custovic A., NAC Manchester Asthma and Allergy Study Group (2004). Early life environmental control: Effect on symptoms, sensitization, and lung function at age 3 years. Am. J. Respir. Crit. Care Med..

[77] Pfefferle P.I., Keber C.U., Cohen R.M., Garn H. (2021). The Hygiene Hypothesis—Learning from but Not Living in the Past. Front. Immunol..

[78] Ege M.J., Mayer M., Normand A.C., Genuneit J., Cookson W.O., Braun-Fahrländer C., Heederik D., Piarroux R., von Mutius E., GABRIELA Transregio 22 Study Group (2011). Exposure to environmental microorganisms and childhood asthma. N. Engl. J. Med..

[79] Platts-Mills T.A., Carter M.C. (2017). Asthma and indoor exposure to allergens. N. Engl. J. Med..

[80] Haahtela T., Holgate S., Pawankar R., Akdis C.A., Benjaponpitak S., Caraballo L., Demain J., Portnoy J., von Hertzen L., WAO Special Committee on Climate Change and Biodiversity (2013). The biodiversity hypothesis and allergic disease: World allergy organization position statement. World Allergy Organ. J..

[81] Indolfi C., D’Addio E., Bencivenga C.L., Rivetti G., Bettini I., Licari A., Manti S., Mori F., Miraglia Del Giudice M., Klain A. (2023). The Primary Prevention of Atopy: Does Early Exposure to Cats and Dogs Prevent the Development of Allergy and Asthma in Children? A Comprehensive Analysis of the Literature. Life.

[82] Ober C., Yao T.-C. (2011). The genetics of asthma and allergic disease: A 21st century perspective. Immunol. Rev..

[83] Moffatt M.F., Kabesch M., Liang L., Dixon A.L., Strachan D., Heath S., Depner M., von Berg A., Bufe A., Rietschel E. (2007). Genetic variants regulating ORMDL3 expression contribute to the risk of childhood asthma. Nature.

[84] Deng J., Tang H., Zhang Y., Yuan X., Ma N., Hu H., Wang X., Liu C., Xu G., Li Y. (2023). House dust mite-induced endoplasmic reticulum stress mediates MUC5AC hypersecretion via TBK1 in airway epithelium. Exp. Lung Res..

[85] Hinds D.A., McMahon G., Kiefer A.K., Do C.B., Eriksson N., Evans D.M., St Pourcain B., Ring S.M., Mountain J.L., Francke U. (2013). A genome-wide association meta-analysis of self-reported allergy identifies shared and allergy-specific susceptibility loci. Nat. Genet..

[86] Wood L.G., Garg M.L., Gibson P.G. (2011). A high-fat challenge increases airway inflammation and impairs bronchodilator recovery in asthma. J. Allergy Clin. Immunol..

[87] Dogra S., Kuk J.L., Baker J., Jamnik V. (2011). Exercise is associated with improved asthma control in adults. Eur. Respir. J..

[88] Klain A., Giovannini M., Pecoraro L., Barni S., Mori F., Liotti L., Mastrorilli C., Saretta F., Castagnoli R., Arasi S. (2024). Exercise-induced bronchoconstriction, allergy and sports in children. Ital. J. Pediatr..

[89] Eisner M.D., Yelin E.H., Katz P.P., Earnest G., Blanc P.D. (2002). Exposure to indoor combustion and adult asthma outcomes: Environmental tobacco smoke, gas stoves, and woodsmoke. Thorax.

[90] Lewis G., Milnes L., Adams A., Schwarze J., Duff A. (2023). Influences on indoor environmental trigger remediation uptake for children and young people with asthma: A scoping review. Health Expect..

[91] Baxi S.N., Phipatanakul W. (2010). The role of allergen exposure and avoidance in asthma. Adolesc. Med. State Art. Rev..

[92] Custovic A., de Moira A.P., Murray C.S., Simpson A. (2023). Environmental influences on childhood asthma: Allergens. Pediatr. Allergy Immunol..

