# Correction: Klain et al. The Prevention of House Dust Mite Allergies in Pediatric Asthma. *Children* **2024**, *11*, 469

**DOI:** 10.3390/children11070774

**Published:** 2024-06-27

**Authors:** Angela Klain, Antonio Andrea Senatore, Amelia Licari, Francesca Galletta, Irene Bettini, Leonardo Tomei, Sara Manti, Francesca Mori, Michele Miraglia del Giudice, Cristiana Indolfi

**Affiliations:** 1 Department of Woman, Child and General and Specialized Surgery, University of Campania ‘Luigi Vanvitelli’, 80138 Naples, Italy; angela.klain@studenti.unicampania.it (A.K.); cristiana.indolfi@policliniconapoli.it (C.I.); 2 Department of Clinical, Surgical, Diagnostic, and Pediatric Sciences, University of Pavia, 27100 Pavia, Italy; antonioandrea.senatore01@universitadipavia.it (A.A.S.); amelia.licari@unipv.it (A.L.); 3 Pediatric Clinic, Fondazione IRCCS Policlinico San Matteo, 27100 Pavia, Italy; 4 Pediatric Unit, Department of Human Pathology in Adult and Developmental Age ‘Gaetano Barresi’, University of Messina, 98122 Messina, Italy; francygall.92@gmail.com (F.G.); smanti@unime.it (S.M.); 5 Pediatric Unit, IRCCS Azienda Ospedaliera-Universitaria di Bologna, 40138 Bologna, Italy; irene.bettini@studio.unibo.it; 6 Allergy Unit, Meyer Children’s Hospital, IRCCS, 50139 Florence, Italy; leonardo.tomei@unifi.it (L.T.); francesca.mori@unifi.it (F.M.)

There was an error in the original publication [1]. There is a resemblance between a certain part of the text in the published paper and the review article “The Role of Dust Mites in Allergy” by Miller, J.D. A correction has been made to Section 2, Understanding HDM Allergies and Asthma, in the fourth paragraph:

Chitin is a component of the exoskeletons of shrimp, crustaceans, insects, worms, and dust mites. This protein is highly allergenic, triggering the activation of the T2 immune system and the production of two asthma-related molecules: acidic mammalian chitinase (AMC) and the chitinase-related protein YKL-40 [1,22]. These proteins serve as markers of severe asthma in sensitized individuals. Additionally, endotoxins present in dust mite faeces act as allergens, inducing inflammation and hyper-reactivity in the bronchial pathways [1,23].

The authors state that the scientific conclusions are unaffected. This correction was approved by the Academic Editor. The original publication has also been updated.
